# Toward innovative approaches for exploring the mechanically regulated tumor-immune microenvironment

**DOI:** 10.1063/5.0183302

**Published:** 2024-02-21

**Authors:** Maria Kalli, Triantafyllos Stylianopoulos

**Affiliations:** Cancer Biophysics Laboratory, Department of Mechanical and Manufacturing Engineering, University of Cyprus, Nicosia, Cyprus

## Abstract

Within the complex tumor microenvironment, cells experience mechanical cues—such as extracellular matrix stiffening and elevation of solid stress, interstitial fluid pressure, and fluid shear stress—that significantly impact cancer cell behavior and immune responses. Recognizing the significance of these mechanical cues not only sheds light on cancer progression but also holds promise for identifying potential biomarkers that would predict therapeutic outcomes. However, standardizing methods for studying how mechanical cues affect tumor progression is challenging. This challenge stems from the limitations of traditional *in vitro* cell culture systems, which fail to encompass the critical contextual cues present *in vivo*. To address this, 3D tumor spheroids have been established as a preferred model, more closely mimicking cancer progression, but they usually lack reproduction of the mechanical microenvironment encountered in actual solid tumors. Here, we review the role of mechanical forces in modulating tumor- and immune-cell responses and discuss how grasping the importance of these mechanical cues could revolutionize *in vitro* tumor tissue engineering. The creation of more physiologically relevant environments that better replicate *in vivo* conditions will eventually increase the efficacy of currently available treatments, including immunotherapies.

## FORCES EXERTED ON CELLS WITHIN THE TUMOR MICROENVIRONMENT

A solid tumor constitutes a complex and dynamic tissue, comprised not only of cancerous cells but also of immune and stromal cells, all embedded within an extracellular matrix (ECM). ECM undergoes alterations that contribute to the tumor's mechanical characteristics, such as elevated matrix stiffness, which facilitates tumor expansion and persistence within the host organ. Increased ECM stiffness hinders the penetration of drugs and immune cells into the tumor interior,^1^ while it can activate mechanotransduction pathways in several cellular components of TME including fibroblasts, cancer cells, and immune cells.^2,3^ This, in turn, regulates different cellular responses, such as cytoskeleton organization, migration, and proliferation, as well as the activation state of fibroblasts and immune cells, which eventually promote tumor progression and therapy resistance.^4–7^

The expansion of a rigid tumor within the confined space of the host tissue causes the generation of mechanical forces both intratumoral and at the interface of the tumor with the neighboring host tissue. These mechanical forces—which can be both compressive and tensile—are transmitted on cellular membranes residing within the tumor microenvironment (TME), leading to resistance against cellular deformation and eventually producing intracellular stress (i.e., force per unit area).^2,8–10^ The stress coming from the solid constituents of the TME is called “solid stress.”^11,12^ Solid stress exerts a dual role within the TME, it can directly regulate cellular responses through mechanotransduction, and indirectly compress the tumor vasculature, including blood and lymphatic vessels. Recently, ECM stiffness and solid stress have been considered as two distinct biomechanical abnormalities in the TME.^13^ Stiffness is a material property describing the extent to which a material resists deformation in response to an applied force, while solid stress is a force per unit area. In solid tumors, the stiffness is mainly determined by ECM composition and organization and by cellular density, while solid stress arises by the sum of the physical forces exerted during tumor growth. However, both biomechanical abnormalities impair the delivery of oxygen, nutrients, immune cells, and drugs into the tumor interior.^10^ The correlation of ECM stiffness with solid stress is reviewed in detail elsewhere.^12^

The fluid components of the TME exert additional mechanical forces, namely, fluid stresses, most often of lower magnitude than solid stress. These stresses are derived either from interstitial fluid flow that gives rise to interstitial fluid pressure (IFP) and shear stresses on structural components within the TME, or from the fluid shear stress induced by the dynamic flow of blood and lymphatic fluid against vessel walls.^10^ Like solid stress, fluid stresses act at the cellular level to regulate cellular responses and at the tissue level can compromise the delivery of vital entities within the tumor, such as nutrients, immune cells, and therapeutic agents.

As the effect of matrix stiffness is extensively being studied and several *in vitro* models that mimic this biomechanical abnormality have widely been employed, we focused here on elucidating the advancements in research on how solid and fluid stresses affect cancer cells. With the current emphasis on using immunotherapy to treat cancer, we also highlight studies that provide evidence for mechanically driven cancer immunity, which should increase the efficacy of these therapeutic strategies. Finally, we propose innovative experimental approaches that could be integrated into future investigations to enhance the efficacy of currently available treatments.

## THE EFFECT OF MECHANICAL FORCES ON CANCER CELLS: A TIMELINE

### The role of solid stress in tumor progression

#### Solid stress-induced effect on cancer cell proliferation

**FIG. 1.**
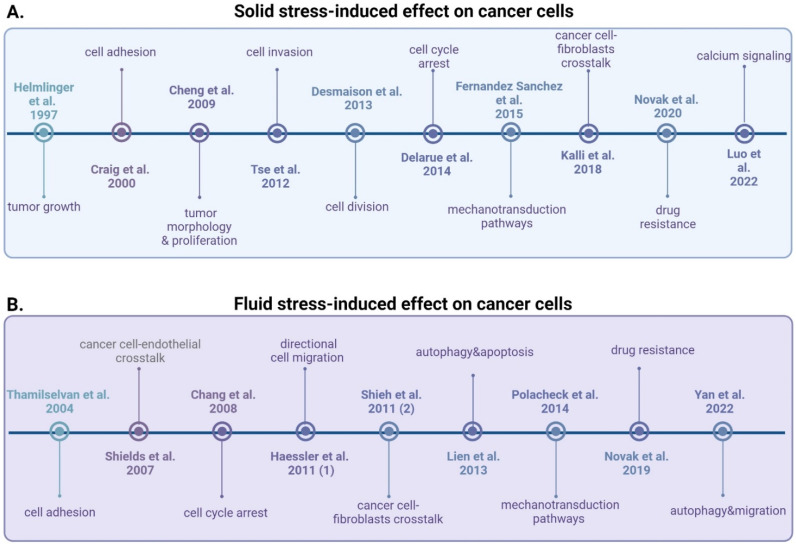
Timeline of the key studies that focus on the effect of solid (a) and fluid stress (b) on cancer cells. Created with BioRender.com.

The influence of mechanical stresses on cell shape and the underlying cellular mechanisms that respond to these stresses was elucidated in the early 1990s^14–16^ [Fig. 1(a)]. These studies unveiled the significant role of mechanical forces in regulating cellular movement, proliferation, apoptosis, and the acquisition of stem cell characteristics.^17–20^ Later, in 1997, the seminal work by Helmlinger *et al.* demonstrated and quantified the emergence of solid stress on cancer cells by studying the growth of tumor spheroids within an agarose matrix of varying concentration. This *in vitro* system, devoid of any other biochemical influence (e.g., ECM constituents), was shown to hinder the progression of breast and colon tumor spheroids.^21^ Building upon these findings, Cheng *et al.* employed tumor spheroids of breast cancer cells using a similar setup, highlighting that space confinement, the major contributor for solid stress generation in the TME, hindered spheroid growth.^22^ These observations were further supported by other studies showing that the division of colon cancer cells grown as spheroids within a Dextran matrix of increasing concentrations was governed by mechanical stress.^23^ Later, it was shown that mechanical stress developed within a synthetic ECM-mimicking polymer, polydimethylsiloxane (PDMS), disrupted the mitotic division of colorectal tumor cells embedded as spheroids,^24^ which as revealed by subsequent studies, was triggered by p27-mediated cell cycle arrest.^25^

#### Solid stress-induced effect on cancer cell migration and invasion

Beyond the impact on cancer cell proliferation, mechanical stress is well known for its role in promoting the metastatic potential of cancer cells. Initial indications were discovered in 2000, where a mechanical pressure was applied on colon cancer cells using an airtight box equipped with inlet and outlet valves. This custom-made approach demonstrated an increase in cell adhesion to the matrix through the activation of integrins.^26^ A different, custom-made device was used to apply compressive force on breast and glioma cancer cells growing as individual cells in a 3D matrix composed of agarose. This study highlighted the alteration of a metastasis-related gene expression profile under compression, consequently affecting the migratory potential of cells.^27^ In line with these findings, the seminal work by Tse *et al.* played a pivotal role in elucidating the significance of mechanical forces in cell migration. In this study, they introduced a 2D transmembrane pressure device to apply a predefined compression on a cell monolayer seeded on a porous membrane of the inner chamber of a transwell insert, which allows the efficient transmission of nutrients and oxygens from the outer chamber toward cells, preventing their transmigration. This study demonstrated that the application of mechanical pressure directly stimulates the migration of breast cancer cells by enhancing actomyosin contractility and fostering leader cell formation.^28^ This was the first study providing straightforward evidence of a compression-induced migration using live cell imaging. The leader cell formation caused by compression was later found to be facilitated by mechanically induced activation of Notch signaling.^29^ Moving deeper into the mechanically driven signaling pathway activation, compression applied in 2D was found to enhance interleukin-6 (IL6)-induced epithelial-to-mesenchymal transition (EMT) and stemness of renal cancer cells through Akt/GSK-3β/β-catenin pathway activation.^30^ Our lab further indicated that mechanical solid stress, applied using a similar setup as in Tse *et al.*'s work, facilitated interactions between fibroblasts and cancer cells, leading to increased migratory ability of the cancer cells.^31^ Subsequently, we revealed that PI3K/Akt signaling activation in pancreatic cancer cells was necessary to increase their migratory potential under compressive solid stress, like what has been observed in renal cancer cells.^32^ This compression-induced effect has also been observed in glioblastoma cancer cells, in which case it was mediated through MEK1/ERK1 signaling activation^33^ and micro-RNA (miRNA) and mRNA interactions.^34^ Investigations involving hepatocellular carcinoma revealed that mechanical stretching, facilitated by an electromagnetic device, leads to the activation of a mechanotransduction mechanism involving RhoA and Rac1 genes, both implicated in cytoskeleton organization.^35^ Mechanical force applied using atomic force microscopy (AFM) revealed that it induces Cl^−^ efflux, which was correlated with the metastatic potential of breast cancer cells,^36^ while in a different approach, mechanical pressure was applied on colon cancer cells i*n vivo* using magnetic beads, which activated β-catenin signaling altering gene expression, thereby promoting tumor progression.^37^ Similar *in vivo* compression approach has also been employed in brain tissues, to reveal that compressive solid stress reduced tumor perfusion and promotes tumor progression.^38^ Collectively, these studies demonstrated that mechanical compression promotes the metastatic ability of cancer cells, mainly through the activation of PI3K/Akt/β-catenin signaling axis that regulates gene expression, cytoskeletal changes, and actomyosin contractility.

#### Other solid stress-induced cellular responses

While there has been a considerable focus on understanding the effects of mechanical stress on cancer cell migration and proliferation, its effect on other cellular responses that also drive tumor progression as well as drug resistance is still under investigation. Breast cancer cell spheroids embedded in an agarose matrix showed reduced response to the chemotherapeutic agent doxorubicin.^39^ Compression applied to breast cancer cells encapsulated in alginate beads and embedded within an agarose matrix was shown to affect angiogenesis by inducing overexpression of vascular endothelial growth factor (VEGFA) through chromatin modifications.^40^ In line with these results, confinement of breast tumor spheroids led to resistance of breast cancer cells to cisplatin, which acquire stemness, migrative and proliferative capabilities, as well as increased contractility and proteolytic ability through production of matrix metalloproteinases (MMPs).^41^ Hydrostatic pressure- and growth-induced compression were found to regulate the expression of the cytoskeleton regulator, cdc42, which subsequently affect the proliferation, elongation, and chemoresistance of cells growing in 3D hydrogels within a bioreactor system.^42^ In another novel approach, mathematical modeling and tumor spheroids were utilized to reveal that confinement could reduce the proliferation of pancreatic tumor cells and increase drug resistance through ERK signaling activation.^43^ Mechanical stresses also drive nuclear softening, which as shown in healthy cells is necessary for their protection against external mechanical forces.^44,45^ Nuclear softening is linked with chromatin alterations and epigenetic modifications and, hence, it can promote gene expression alterations. Furthermore, compression applied on HeLa cells growing within alginate capsules, induced the activation of autophagy and increased their invasive capabilities through p38 MAPK signaling activation,^46^ while additional studies have discovered the mechanosensitive Piezo channels that orchestrate calcium signaling to regulate the mechanically driven tumor progression.^47,48^ A summary of recent studies providing evidence of a direct solid stress-induced molecular effect on cancer cells and the experimental setup used in each case are shown in Table I. The experimental setups mainly include (i) a transmembrane pressure device for the application of predefined compressive force on a cell monolayer in 2D and (ii) the 3D growing of cells either as single cells or as spheroids within a hydrogel or microcapsules. In most cases, a single cancer cell line was used.

**TABLE I.** Summary of recent studies focused on the direct effect of mechanical forces on cancer cells.

Cancer type	Experimental setup	Effect on cancer cells	Reference
The effect of solid stress on cancer cells			
Pancreatic	2D transmembrane pressure device/cell monolayer	Fibroblast-induced migration of cancer cells *via* GDF15	31
Glioblastoma	2D transmembrane pressure device on cell monolayer/3D spheroid growth in agarose hydrogel	MEK/ERK signaling activation → increased migration	33
Pancreatic	2D transmembrane pressure device/cell monolayer	PI3K/Akt/CREB signaling activation → increased migration	32
Hepatocellular	Electromagnetic device	RhoA/Rac1 activation → cytoskeletal organization	35
Glioblastoma	2D transmembrane pressure device/cell monolayer	miRNA/mRNA interactions → increased migration	34
Ovarian	Single cells growing in a 3D hydrogel bioreactor system	Increased Cdc42 expression → cell proliferation, invasion, and chemoresistance	42
Pancreatic	Spheroids growing in 3D hydrogel	Drug resistance	43
Glioblastoma	*In vivo* compression device	Reduced tumor perfusion and increased tumor progression	38
Breast	AFM-induced mechanical force	Increased Cl^−^ efflux → increased metastatic potential	36
Breast	Spheroids growing in 3D hydrogels/single cells growing in microcapsules	Increased cancer cell stemness, migration and cisplatin resistance	41
Breast	2D transmembrane pressure device/cell monolayer	Piezo1 signaling activation → increased invasion and matrix degradation	48
The effect of fluid stress on cancer cells			
Ovarian	2D rotator-induced shear stress/cell monolayer	Decreased cell viability, increased spheroid formation, cytoskeletal alterations → increased invasion	65
Hepatocellular	2D parallel plate flow chamber/cell monolayer	Integrin/FAK/Rho GTPases signaling pathway activation → increased migration	62
Hepatocellular	2D parallel plate flow chamber/cell monolayer	FAK/ERK signaling activation → decreased stiffness and increased migration of cancer stem cells	63
Breast	3D single cells growing in hydrogel bioreactor generating shear stress	Increased cell motility, proliferation, PLAU gene expression and chemoresistance	68
Pancreatic	3D microfluidic interstitial flow model	Inhibition of cell proliferation, increase in ABC drug transporter gene expression → chemoresistance	69
Breast	Microfluidic device generating hydrostatic pressure	Increase in ABC drug transporter gene expression → chemoresistance	70
Hepatocellular	2D parallel plate flow chamber/cell monolayer	RhoA-YAP1-autophagy pathway activation → migration of cancer stem cells	64
Liver	2D parallel plate flow chamber/cell monolayer	Shear stress-induced exosome secretion by cancer cells to activate stellate cells through PI3K signaling activation	55

### The role of interstitial flow-induced shear stress in tumor progression

#### Shear stress-induced effect on cancer proliferation and apoptosis

Cells within the TME are exposed to hydrostatic pressure and interstitial fluid flow-induced shear stress that could act synergistically with solid stress to regulate cellular responses. To mimic flow-induced shear stress *in vitro*, experimental setups usually employ a parallel flow chamber, a microfluidic device or a simple stirring of suspended cells. Similar to solid stress, shear stress was found to impair cell cycle progression as revealed by alterations in the expression of several cyclins, including cyclin B1 and p21, through integrin-mediated activation of Smad-signaling.^49^ Later, the shear stress-induced effect was shown to be mediated through the activation of p38 MAPK signaling and autophagy, leading to the induction of apoptosis in hepatocellular carcinoma and osteosarcoma cells.^50^

#### Shear stress-induced effect on cancer cell migration and invasion

The effect of shear stress on the motility as well as the direction of migration has been revealed in several cancer cell lines, while different signaling pathways were shown to be involved. In colon cancer cells, Src signaling was activated to induce actin reorganization and increase the adhesion of cells to collagen I substrate.^51^ Interstitial fluid flow applied on breast cancer cells seeded within a 3D collagen I scaffold resulted in β1-integrin activation and vinculin-, FAK-, F actin-, and paxillin-dependent protrusion directing the migration of the cells.^52^ This flow-induced directional migratory stimulus was found to be also mediated by the transmembrane receptor CCR7, which similar to β1-integrin, leads to FAK signaling pathway activation.^53^

In addition to these mechanotransduction pathways, the PI3K pathway was shown to be activated in breast cancer cells exposed to shear stress^54^ and be implicated in cancer cell-cancer associated fibroblast (CAF) interactions.^55^ In line with these findings, interstitial flow applied on a coculture system composed of breast cancer cells and fibroblasts promoted fibroblast migration toward collagen fibers through increased transforming growth factor-β1 (TGFβ) activation and collagen degradation, which eventually enhanced tumor cell invasion.^56^ Regarding breast cancer-endothelial cell interactions, shear stress mediates the induction of an autologous secretion of ligands that direct breast cancer cells toward lymphatic endothelial cells.^57,58^

The interstitial flow-enhanced motility was also observed for glioma^59^ and hepatocellular carcinoma cells^60^ using a 3D invasion assay.^61^ The flow-induced hepatocellular cancer cell invasion to liver is mediated through the activation of CXCR4/CSCL12 signaling and MEK/ERK pathway^60^ like the compression-induced activation of MEK1/ERK1 signaling in glioblastoma cancer cells.^33^ Further work suggested that the migration of hepatocellular and liver carcinoma cells is increased as a response to flow-induced shear stress through the activation of integrin/FAK/Rho GTPase signaling cascade similar to what has been observed in breast cancer cells.^62,63^ In support to these findings, fluid shear stress induces liver cancer stem cell proliferation and increases spheroid-formation abilities and migration via RhoA-YAP1-autophagy pathway.^64^ Similar results were observed for ovarian cancer cells cultured on a rotator that mimics the interstitial fluid flow. Cells exhibited reduced viability, increased ability for spheroid formation, and altered cytoskeletal organization upon exposure to shear stress.^65^ Subsequently, glycocalyx components such as hyaluronan, which cover cancer cell surface, were revealed to sense and transmit shear forces to increase the motility of renal carcinoma cells by altering MMP, CD44, and integrin gene expression levels.^66^ Hydrostatic pressure applied on lung cancer cells, increased their migratory and invasive potential, cell volume, filopodial number, migration-related gene expression levels, as well as phosphorylation of caveolin-1 and ERK1/2.^67^

#### Shear stress-induced effect on cancer cell chemoresistance

Apart from the effect of shear stress on cell proliferation and migration, breast cancer cells growing in a 3D hydrogel and exposed to shear stress stimulus had significantly higher motility, were more proliferative, and showed paclitaxel resistance.^68^ In line with these findings, pancreatic and breast cancer cells exposed to fluid flow exhibited reduced proliferation and increased gemcitabine^69^ and doxorubicin^70^ resistance, respectively, which was mediated by alterations in multidrug resistance-related gene expression.

The key studies focused on the effect of fluid flow and hydrostatic pressure on cancer cells are chronologically shown in Fig. 1(b). A summary of the most recent studies and the experimental setup used in each case is shown in Table I. In most cases, a parallel flow chamber was used to simulate fluid flow-induced shear stress, and a single cancer cell line cultured as a 2D cell monolayer was employed.

While many studies have looked at how mechanical forces affect cancer cells and several strategies have been developed to block these mechanisms at the cellular and tissue level,^7,71^ we still have limited knowledge about how these forces impact immune cells in the TME. As current directions move on the use of immunotherapy in cancer patients, understanding these mechanisms holds the key to increase its effectiveness and reduce toxicities associated with chemotherapy-based therapeutic strategies.

## HOW INTRATUMORAL MECHANICAL FORCES AFFECT IMMUNE RESPONSES?

**FIG. 2.**
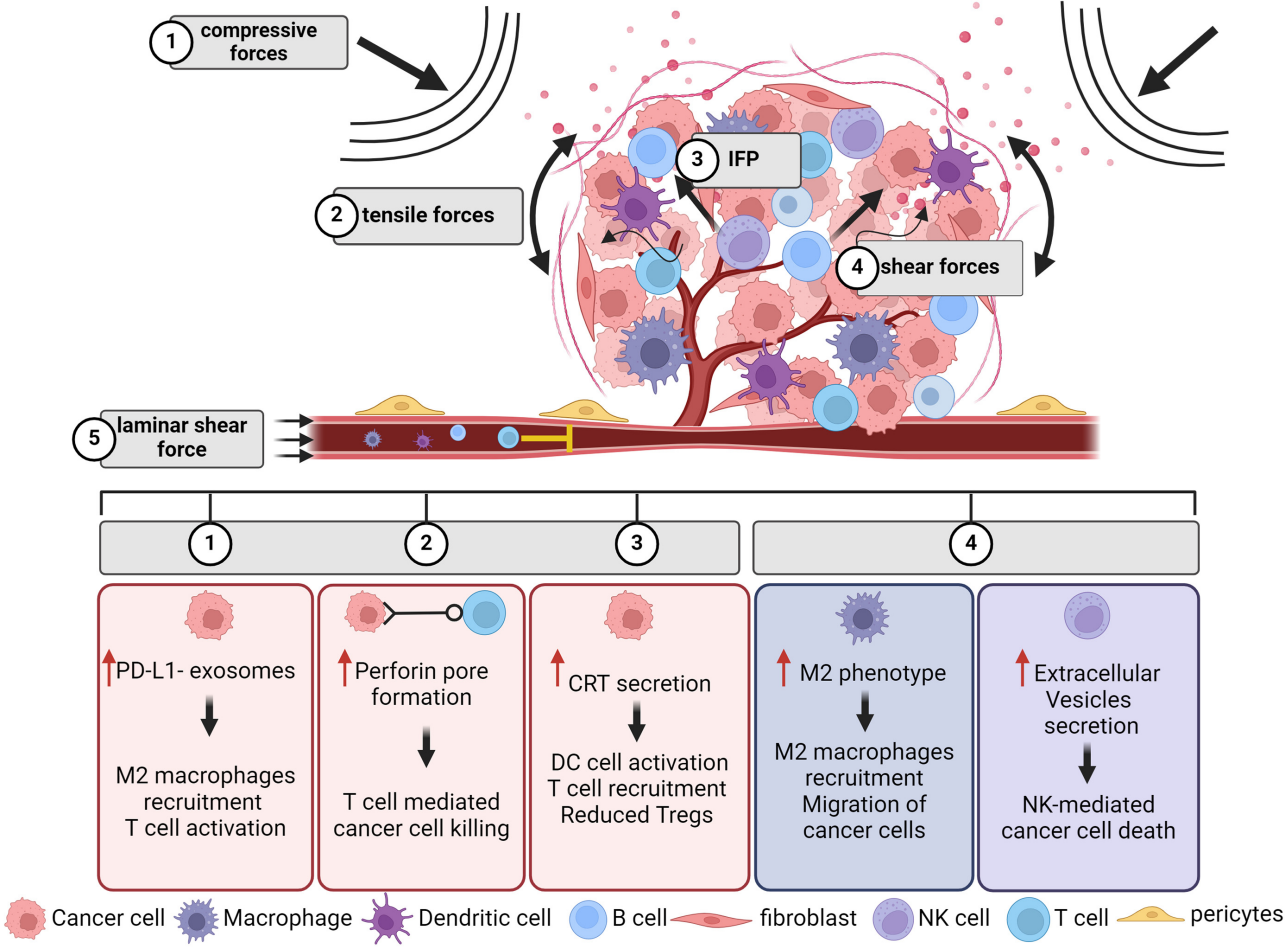
The biomechanically altered TME affects the function of both cancer and immune cells. As tumor grows within a confined space of the host tissue, mechanical forces are generated. These forces are compressive or tensile and can compress the tumor vasculature, including blood and lymphatic vessels hindering the transport of vital entities, such as immune cells, in the tumor interior. At the same time, the fluid components of the TME exert additional mechanical forces including the interstitial fluid pressure (IFP), laminar shear force, and the interstitial flow that generates shear stresses on structural components within the TME. The effect of these mechanical stimuli on cancer cells and how it is correlated with immune functions are shown in the respective boxes. Evidence of the direct force-induced immune responses and how they affect tumor progression are shown in the respective boxes for (i) T cells; (ii) macrophages; and (iii) Natural killer cells. Create with BioRender.com.

Under the influence of mechanical cues, cancer cells also interact with diverse types of immune cells. Among the key immune players are T- and B-cells, macrophages, natural killer (NK) cells, and dendritic cells (DCs). Each cell type relies on physical interactions with other cells to initiate their response; thus, it is possible that mechanical forces play a crucial role in their activation process during tumor progression. Nevertheless, only a few studies have focused on the link between the physical aspects of the TME and cancer immunity. As all cells in the TME are exposed to different mechanical forces, including compressive, tensile, and shear forces (Fig. 2), it is reasonable to study how each cell type responds to these mechanical stimuli, and how altogether they synergize to regulate tumor progression. All studies focused on how mechanical forces affect immune cells and cancer–immune cell interactions are summarized in Table II.

**TABLE II.** Summary of studies focused on the effect of mechanical forces on immune responses.

Cell type	Mechanical stimuli	Mechanotransduction pathway	Immune response	Effect on tumor immunity?	References
T cells	Pulling and shear force (single cell level)		TCR conformational changes → T cell activation		74 and 75
	Increased stiffness	YAP signaling activation	Suppression of metabolic reprogramming of T cells		78
Tregs	Hyaluronan-induced force	HA-CD44 cross-linking → TCR activation	Increase of IL-2 secretion → maintenance of Treg viability and function		79
B cells	Rupture force (single cell level)		BCR activation		80
Macrophages	Mechanical stretching		IL-6, IL-8, MMP9 secretion → tissue damage repair		82
		RhoA/ROCK/NF-κΒ signaling activation	M1 polarization and activation		83
	Cyclic hydrostatic pressure	Piezo1/JNK/Jun signaling activation	Pro-inflammatory response		88
	Increased stiffness	YAP activation	Inhibition of inflammatory activation		85
		Piezo1/IFNγ/LPS signaling and NFκB activation	Macrophage polarization and increased activation		86
	Interstitial flow	Integrin/SRC signaling activation	M2 polarization → increased macrophage migration rate and migration against flow	1. Increased cancer cell motility	89
				2. M2 macrophages recruitment toward tumor	

NK cells	Shear forces		Secretion of extracellular vesicles containing cytotoxic proteins	Increased cancer cell death/tumor suppression	91
	Mechanical force (single cell level)		Conformational changes of NKG2D ligand → Activation of NK cells		92
DC cells	Shear forces		Increased directness of DC migration and activation		93
		NF-κB-, c-Fos signaling activation	Increased DC activation		94
Cancer cells	Mechanical tension		Increased perforin-induced pore formation	Increased T cell-mediated cancer cell killing	95
	Hydrostatic pressure		Increased secretion of CRT	Increased DC activity and T cell recruitment	97
				Decreased number of Tregs	
	Mechanical compression		Compression induced secretion of PD-L1-containing exosomes	Increased number of M2 macrophages and activated T cells	98

### Force-induced T cell responses

Starting from T cells, their major role in the TME is to recognize cancer-specific antigens to get activated and induce cancer cell killing. The activation is facilitated by the T cell receptor (TCR), which recognizes antigens presented by major histocompatibility complexes (MHC) that are present on the membrane of antigen presenting cells (APCs).^72^ The interaction of T cells with these antigens activates the anti-tumor cytotoxic T lymphocyte (CTL) response.^72^ Mechanical cues generated in the TME could regulate the function of T cells, which are shown to be sensitive to several biomechanical signals, and they respond by modulating their activation, adhesion, migration, and triggering of immune responses.^73^ In line with this, it has been shown that molecular-scale physical forces (i.e., acting on single cell level) have the potential to induce activation of T cells by facilitating conformational changes of the TCR receptor. These changes further affect the physical interaction of TCR with the CD3 membrane receptor,^74–76^ a complex that is crucial for the activation of T cells. After their activation, cytotoxic T cells secrete enzymes, such as toxic proteases and the pore forming protein perforin into their synapse with cancer cells to promote their killing.^77^ Further research revealed that YAP (YES Associated Protein), which is among the most well-studied mechanical sensors being sensitive to external mechanical forces and stiffness, suppresses the metabolic reprogramming of effector T cells in a stiffness-dependent manner.^78^ While this effect was confirmed during viral infections that heightened the stiffness of lymph nodes, it is possible that in TME, where the stiffness of the ECM is also elevated, it could potentially enhance YAP-induced suppression of T cells. Regulatory T cells (Tregs) are a specialized subset of T cells that maintain immune tolerance and prevent immune reactions. Within the TME, Tregs can suppress anti-tumor immune responses, enabling tumor immune escape and progression. This is mediated by impairing cell–cell contact, expression of surface molecules, and secretion of cytokines, which have been shown to be regulated, at least in part, by the presence of hyaluronic acid in the ECM.^79^

### Force-induced B cell responses

Like T cells, B lymphocytes use B cell membrane receptors (BCRs) to sense antigens, a process that can be affected by physical cues. While research on this mechanism is limited, it was shown that the cytoplasmic IgG tail of the BCR senses low-level mechanical forces to promote the enrichment of this receptor with the phosphatidylinositol (PI) (4,5) biphosphate phospholipid facilitating BCR activation.^80^ Whether this mechanism is regulated in the TME and how it affects cancer immunity has not yet been reported.

### Force-induced macrophage responses

In contrast with T- and B-cells, macrophages can either promote or inhibit tumor growth and progression. Pro-inflammatory or M1 macrophages act as tumor killers, while anti-inflammatory or M2 macrophages support tumor growth and angiogenesis.^81^ Macrophages respond to external mechanical cues, such as mechanical stretching and tension, by a dynamic switching between the M1 and M2 phenotypes. In line with this, Pugin *et al.* showed that mechanical stretching, as observed during normal ventilation in lungs, activates macrophages to secrete proinflammatory cytokines, IL-6 and IL-8, and MMP9 to repair tissue damage.^82^ Mechanical stretching also enhances M1 polarization, which is linked to their activation, through the RhoA/ROCK/NF-κB signaling pathway.^83^ However, whether these mechanisms are activated in TME is not defined yet. As cells experience mechanical stretching in the TME, it is possible that M1 macrophages get activated and acquire increased anticancer effects during tumor growth. However, the M2 tumor-associated macrophages usually dominate over the M1 subtype in tumors, which is associated with poor prognosis.^84^ Matrix stiffness, which is a prominent mechanical cue in TME, has been suggested to be implicated in macrophage polarization and activation states through the activation of YAP^85^ and Piezo1 channels.^86^ Vice versa, tumor associated macrophages were found to promote ECM stiffening and, hence, cancer cell migration through the secretion of collagen cross-linking enzymes such as lysyl oxidase.^87^ Moreover, macrophages acquire a pro-inflammatory phenotype upon exposure to cyclical hydrostatic pressure.^88^ Additional research revealed that interstitial flow promotes an M2-like phenotype via integrin/Src signaling pathway activation, which results in faster migration rate and an enhanced ability of macrophages to promote the motility of cancer cells. Moreover, fluid flow directs macrophages to migrate against the flow, recruiting the M2 macrophages toward the tumor and eventually promoting tumor progression.^89^

### Force-induced NK cell responses

NK cells play a crucial role in tumor immunity as they directly target tumor cells through the secretion of extracellular vesicles (EVs) or enhance the anti-tumor activity of other immune cells.^90^ Nevertheless, studies on how mechanical cues affect their function in cancer are limited. Wu *et al.* showed that shear forces increase the secretion of NK-derived EVs, which contain cytotoxic proteins, including granzyme A/B and perforin. These EVs were observed to promote melanoma- and liver-cancer cell death *in vitro*, as well as in suppressing melanoma growth *in vivo.*^91^ One of the most relevant immune cell activation receptors is NKG2D, which is considered as a major regulator of NK and T cell activation. While recent studies turned their focus on targeting this receptor, little is known about the effect of mechanical forces on its activation state, which is shown to be influenced at least at the molecular level.^92^

### Force-induced DC cell responses

As expert antigen-presenting cells, DCs play a critical role in initiating and regulating adaptive immune responses. The efficiency of this process affects the extent and effectiveness of anti-tumor immunity and immunotherapies, but how it is regulated by external mechanical signals is vague. It has been revealed that fluid shear stress, simulated to mimic fluid flow in the blood stream, regulates the migration, metabolism, proliferation, and the morphology of DCs and increased the expression of activation markers such as MHC class I.^93,94^ As shear stresses in the TME are generated by interstitial fluid flow, it is possible that DC can get activated to promote immune responses toward cancer cells.

### Force-induced effect on immune-cancer cell and immune cell–stroma interactions

Although research investigating the direct effect of mechanical forces on immune cells is still limited, at least regarding tumor progression, several studies show that mechanical stimuli trigger cancer cells to regulate immune functions. Indeed, mechanical tension of cancer cells during their synapse with T cells and increased cell stiffness of repopulating tumor cells were found to increase T cell-mediated death by heightened pore formation on cancer cell membrane, which was induced by perforin accumulation.^95,96^ Increased hydrostatic pressure applied on cancer cells induced the expression of diverse immunogenic cell death proteins, including calreticulin (CRT). The subsequent interaction of these mechanically stimulated cancer cells with DCs led to an increase in their activity (e.g., phagocytosis), recruitment of high numbers of tumor-specific T cells, and to a reduction in the number of Treg cells.^97^ However, the implantation of pre-compressed breast cancer cells in mice led to immunosuppression, as revealed by increased number of M2 macrophages and reduction of active T cells in TME. This effect was mediated by programmed death ligand 1 (PD-L1)-containing exosomes, which were secreted by cancer cells in response to compression.^98^ In line with these findings, connective tissue stretching has been linked with reduced local inflammation and migration of neutrophils; however, this effect has not been observed in tumor tissues yet.^99^ As mentioned in the previous section, solid and fluid stress can activate PI3K/Akt signaling pathway, induce EMT, and promote autophagy in cancer cells. To this end, it has been suggested that when cancer cells undergo EMT, they have increased resistance to apoptosis, CTL-activated death receptor pathways, and elevated PD-L1 expression, and thus could potentially block CTL-induced cancer cell killing upon exposure to mechanical stress.^100–103^ Autophagy, is a physiological process that facilitates the degradation of cellular organelles and molecules, including the MHC-I and granzyme B in response to environmental stress conditions.^104,105^ Thus, it is reasonable to hypothesize that mechanical-stress induced autophagy activation can degrade these molecules to impair T cell-induced and NK cell-induced apoptosis in cancer cells.^102,105^ However, the autophagic process could also promote the degradation of PD-1/PD-L1 immune checkpoint molecules, which has been found to increase T cell-mediated cancer cell killing.^106^ However, whether and how the activation of EMT and autophagy in cancer cells impair immune responses under mechanical stress conditions is yet to be explored.

Mechanical stresses may also affect cancer immunity and immune tolerance indirectly. The dense ECM along with the compressed blood and lymphatic vessels act as a physical barrier to immune cell infiltration, contributing to the formation of an immune-excluded phenotype.^10,11^ IFP also induces flow of tumor cell- and stromal cell-derived immunosuppressive exosomes toward the tumor boundary. These exosomes may recruit immunosuppressive immune cells to fuel tumor progression.^107^ In addition, tumor stroma contains the TGFβ, which is among the most important regulators of the suppression of T cells that is mediated by the PD1 signaling and the activation of Treg cells.^108–110^ Activated Treg cells can also secrete TGFβ, creating a positive feedback loop. When activated, this cytokine also attracts several immune cells such as NK cells and macrophages to the tumor but impairs their anti-tumor effector functions.^109–112^ Mechanical cues in the tumor stroma, including interstitial flow and contractile forces exerted by CAFs can activate TGFβ, rendering immune cells inactive.^113^ In fact, tumor immunity can be regulated by the interaction of different types of immune cells with each other, with CAFs, as well as with endothelial cells. How mechanical cues can trigger these interactions is not fully clarified. For more details about these mechanisms, we refer the readers to several comprehensive reviews.^114–116^

Collectively, mechanical cues generated in the TME can impact cancer immunity, yet the exact molecular mechanisms remain unclear. This interaction is crucial for immune cells, which depend on physical cell–cell contacts to facilitate their functions, particularly their ability to recognize and kill cancer cells. While substantial research has been conducted to explore how mechanical signals affect T cell function, most of these studies have employed only shear stress or molecular-level forces in their experimental setups. In addition, only a few studies have focused on the impact of mechanical forces on tumor–immune cell interactions, which are shown to strongly influence the efficacy of immunotherapies, at least at the tissue level.^117,118^ Consequently, it is critically important to develop innovative experimental tools that replicate the mechanical conditions as normally generated in the TME, using both cancer and stromal cells, including immune cells. These tools are urgently needed to discover new therapeutic markers that could enhance the effectiveness of current treatments.

## COMBINING NOVEL EXPERIMENTAL SETUPS WITH *MULTI-OMICS* TO EXPLORE THE FORCE-INDUCED CANCER PROGRESSION

While extensive research has been made to explore the effect of mechanical forces on tumor cells, studies investigating immune and stromal cell responses as well as their interactions with cancer cells under the influence of mechanical abnormalities of TME are still limited. In this section, we summarize all the experimental setups that have been developed so far and present novel directions in advancing the models that mimic the TME and allow the comprehensive study of the mechanically driven tumor progression *in vitro*. A summary of recent studies employing novel experimental designs, including the advantages and disadvantages of each model are listed in Table III.

**TABLE III.** Recent advancements in the experimental design for studying the effect of solid and fluid stress *in vitro.*

Experimental design		Mechanical stimuli	Type of cell culture	Type of ECM matrix	Cellular/molecular analysis	Results	Pros	Cons	Reference
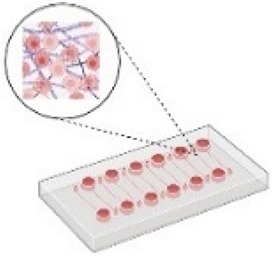	Microfluidic device with separated parallel channels for co-culture of different cell types	Solid stressECM stiffnessShear stress	1. Breast cancer cells2. Monocytes3. Macrophages4. Endothelial cells	Collagen I matrix	1. Cell imaging2. Cell migration3. Angiogenesis (endothelial sprouting)4. Protein and RNA analysis	1. Cancer cells activate monocytes to macrophages2. Cancer cells and fluid flow enhance macrophage activation3. Macrophages and fluid flow increase cancer cell invasion and angiogenesis	1. 3D setup2. Different cell types3. All mechanical stimuli of TME are simulated	1. Matrix composed of Collagen I only2. Cells are not in direct contact	135
		
		
		
	


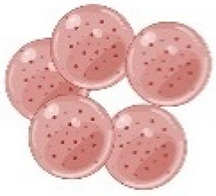	Microcapsules containing tumor spheroids	Solid stress	Breast cancer cells	Alginate-gelatin or Agarose hydrogel	1. Cell imaging2. Cell migration3. Drug sensitivity4. Transcriptomics	1. Gene expression alterations2. Cytoskeletal changes3. Increased migration and stemness4. Drug resistance	1. 3D setup2. Omics data3. Solid stress simulation	1. Cancer cells only2. Absence of physiologically relevant matrix3. Absence of fluid flow	41
	
			
			
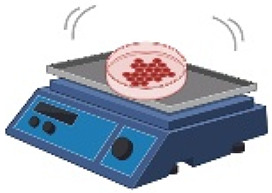	Orbital plate shaker	Shear stress	Ovarian cancer cells	…	1. Cell imaging2. Proteomics	Shear stress regulates proteins related to cell cycle, cell structure and lipid metabolism	1. Omics data	1. 2D culture	150
								2. Cancer cells only	
								3. Absence of physiologically relevant matrix	
								4. Absence of confinement/solid stress	

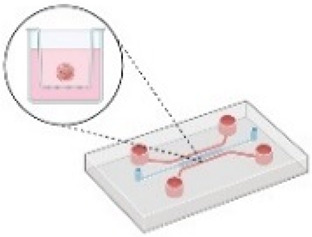	Organ-on-a-chip	1. Solid stress2. ECM stiffness3. Shear stress	1.Neuroblastoma cancer cells2. NK cells	Alginate matrix	1. Cell imaging2. Flow cytometry	Increased migration and activation of NK cells under dynamic fluid flow	1. 3D setup	1. Absence of physiologically relevant matrix	136
							2. Crosstalk of two cell types	2. Absence of additional cell types	
							3. All mechanical stimuli of TME are simulated		

	
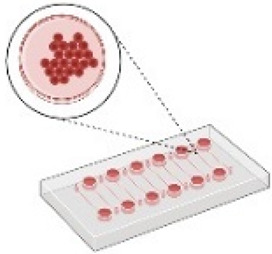	Microfluidic device with separate channels for co-culture of different cell types	1. ECM stiffness2. Shear stress	1. Mesothelioma cancer cells2. Lung fibroblasts	Collagen I matrix	1. Cell imaging2. Gene expression analysis	1. Fluid flow enable the cancer-induced activation and migration of fibroblasts2. Gene expression alterations in activated fibroblasts	1. Easy cell manipulation2. Crosstalk of two cell types	1. 2D culture	138
								2. Cells are not in direct contact	
								3. Absence of additional cell types	
								4. Absence of confinement/solid stress	
	
	

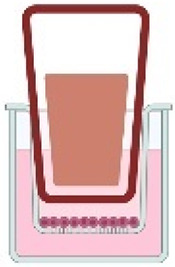	Transmembrane pressure device	Solid stress	Pancreatic cancer cells	…	1. Cell imaging2. Protein and gene expression analysis3. Proteomics	1. Compression activates p38 MAPK-, JNK-, and Rac1/cdc42/Myosin II signaling axes	1. Omics data	1. 2D culture	152
						2. Increased cancer cell migration			
								2. Cancer cells only	
								3. Absence of physiologically relevant matrix	
								4. Absence of fluid flow	


			Breast or pancreatic cancer cells	…	1. Cell imaging	1. PI3K is implicated in compression induced mechano-transduction			151
					2. Protein and gene expression analysis				
					3. Flow cytometry	2. Cell survival under compression could be mediated by autophagy			
					4. Transcriptomics				
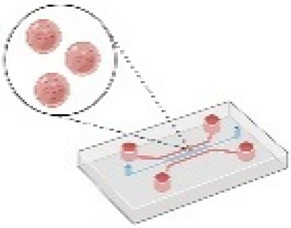	Microfluidic device + microcapsules containing tumor spheroids	1. Solid stress (hydrostatic pressure)2. ECMStiffness	Breast cancer cells only	GelMA matrix	Cell imaging	Spheroid growth is correlated with the mechanical properties of the matrix	3D setup	1. Cancer cells only	131
								2. Absence of fluid flow	
								3. Absence of a physiologically relevant matrix	


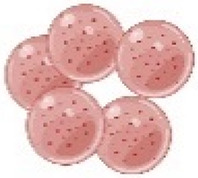 & 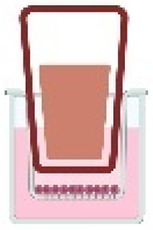	Spheroids embedded in matrix and transmembrane pressure device	1. Solid stress2. ECM stiffness	Melanoma cancer cells	Collagen I matrix	1. Cell imaging2. Protein and gene expression analysis3. Trancriptomics	1. Inhibition of cancer cell proliferation and cell migration2. Organelle and intracellular oxidative stress3. Cancer cell drug resistance	1. 3D setup	1.Cancer cells only	157
								2. Absence of fluid flow	
								3. Matrix composed of collagen I only	
							2. Omics data		
		
	

### From 2D experimental models to organoids-on-a-chip platforms

Animal studies remain the gold standard model for cancer research and preclinical validation of drugs but usually fail to precisely predict human responses in subsequent clinical trials. On the other hand, the traditional *in vitro* culture models offer a simplified but high-throughput method for basic research and drug screening, but they are simplified and cannot fully recapitulate the complex tissue architectures, including the mechanical aspects of the TME.

**FIG. 3.**
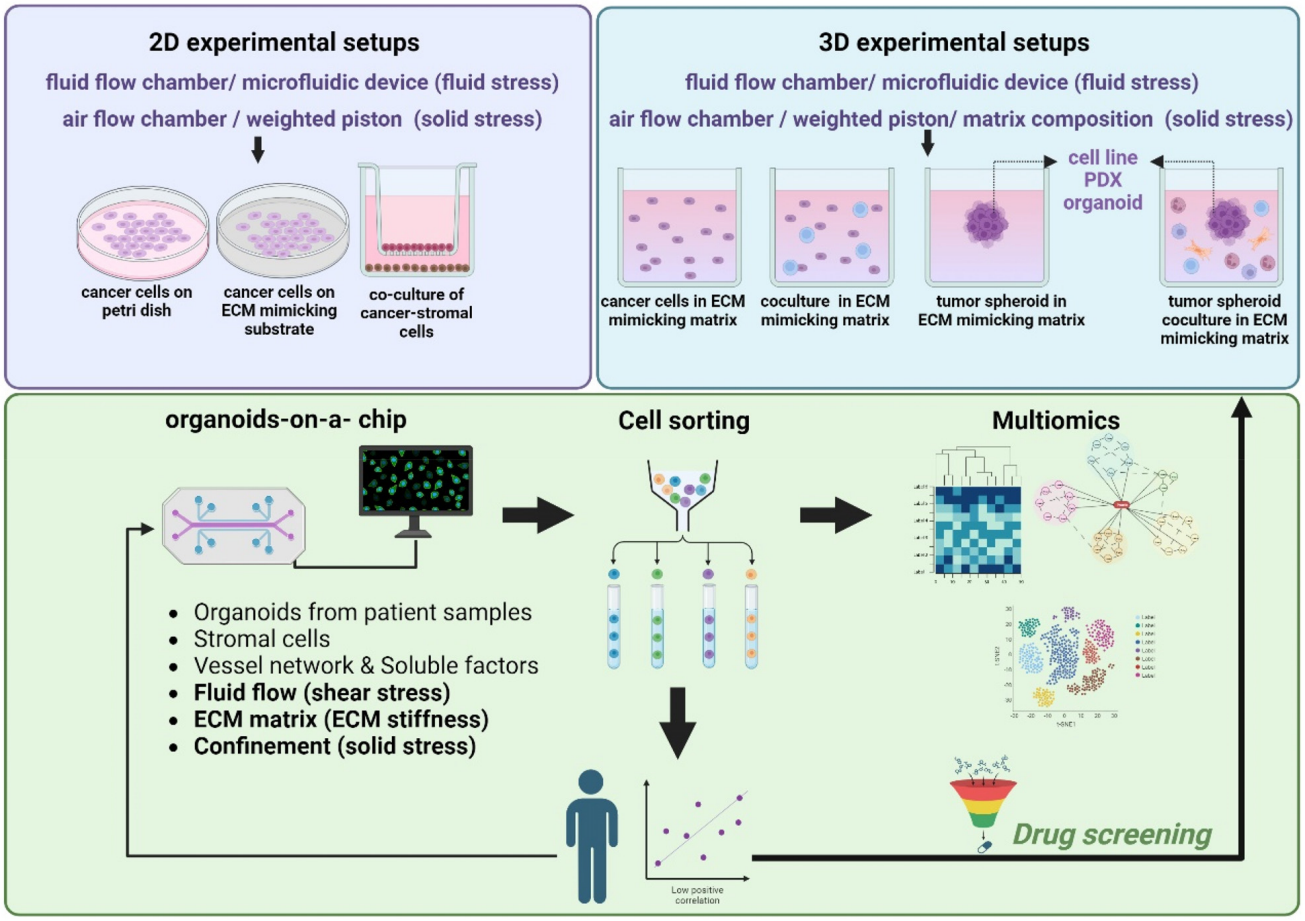
The promise of combining organoids-on-a-chip with multi-omics to target the mechanically driven tumor progression. In the initial stages of studying the mechanically induced tumor progression, simple 2D experimental setups were employed. These setups involved culturing tumor cells on flat surfaces, lacking the complexity of the *in vivo* tumor microenvironment. Experimental setups evolved to include advanced 3D models that are more physiologically relevant. These setups incorporated elements of tissue-like architecture, better simulating the *in vivo* conditions and interactions between cells. To further bridge the gap between *in vitro* and *in vivo* settings, the integration of organoids-on-a-chip platforms was introduced. These microfluidic systems mimic the dynamic physiological conditions found within organs, enhancing the accuracy of mechanical influence studies on tumor progression. Multi-omics approaches, including proteomics and spatial transcriptomics, were then incorporated. These techniques enable the comprehensive analysis of molecular profiles and spatial relationships within the microenvironment, providing insights into mechanotransduction pathways. The combination of advanced platforms and multi-omics techniques facilitates the identification of novel therapeutic biomarkers associated with the mechanically induced tumor progression. To ensure clinical relevance, findings should be validated in patients. This step confirms the applicability of discovered biomarkers in the clinic. The effectiveness of developed therapeutics is tested using the continuum of experimental setups, starting from the simple 2D systems and progressing to the more advanced organoids-on-a-chip platforms. This approach ensures a robust evaluation of treatment strategies across varying degrees of complexity. ECM: Extracellular matrix; PDX: Patient-derived xenograft. Created with BioRender.com.

The 2D experimental models that are usually employed *in vitro* can be divided into three main categories: (i) culture of an established human or murine cancer cell line on traditional petri dishes that are tissue cultured, (ii) co-culture systems that contain two different cell types (e.g., cancer cells and fibroblasts) either in direct contact or separated using different culture chambers, and (iii) culture or co-culture of cells on an ECM mimetic substrate, such as fibronectin or collagen I. Changes in the substrate stiffness or the use of bioreactors, transmembrane pressure, and microfluidic devices have been employed to mimic mechanical cues present in the TME, including fluid flow-induced shear stress, compressive forces, and increased stiffness^12,61^ (Fig. 3). Although these 2D setups are well established and easy to perform, they are difficult to be related to an *in vivo* system or human patients.^119^

Thus, 3D tumor models have been developed and include (i) cancer cell lines cultured as single cells embedded in hydrogels that mimic ECM composition [based on natural materials, e.g., Matrigel, alginate, and collagen I, or synthetic materials, e.g., polyethylene glycol (PEG) and PDMS], (ii) spheroids composed of a single cell line, or (iii) spheroids composed of a mixture of different cell types, such as cancer cells, endothelial cells, and immune cells. The spheroids are usually embedded in 3D ECM mimicking scaffolds as in case (i).^120^ These models have been already used to study the effect of physical aspects of the TME,^12^ but to better recapitulate the native tissues, research efforts were shifted toward developing novel 3D culture systems (Fig. 3), such as patient-derived xenografts (PDX) models. PDXs involve transplanting patient-derived tumor tissues into a matrix in an *in vitro* platform (as shown in Fig. 3) or in immunodeficient mice. These models maintain the heterogeneity and complexity of human tumors and can be used to study tumor behavior such as cell migration and proliferation, biomarker discovery, and drug sensitivity. However, the success rate of their *in vitro* growth is low, their culture is time consuming and has higher relative costs. Moreover, PDX models lack the physiological human microenvironment and its contained factors, such as the physical properties of the ECM and the vascular networks.^121^ To this end, *tumor organoids (or tumoroids*) offer a promising alternative to bridge the gap between cell lines and *in vivo*. They provide a 3D structure grown from cells derived from a needle biopsy or from patient's tissue or mice tumor tissue after surgical resection and are more realistic in recapitulating the *in vivo* TME. The first organoids from patient-derived prostate cancer were developed in 2014^122,123^ followed by pancreatic tumor organoids developed in 2015.^124^ When these organoids are co-cultured with immune and endothelial cells, it could offer an ideal tool for the study of tumor growth, invasion, and response to treatments in physiologically relevant conditions.^125^

Although extensive progress on the development of tumor models has been made, the format of the experimental setup chosen to study the effect of mechanical cues on tumor progression is still a challenge. For example, to study the effect of a predefined compressive force on cancer cells, a 2D cell culture system composed of a single type of cells and the use of a transmembrane pressure device^28^ would be sufficient. If the goal is to test its effect in a heterogeneous TME, a 3D spheroid model composed of cancer cells, cancer stem cells, immune cells, and different stromal cells, such as fibroblasts and endothelial cells embedded in an ECM-mimicking matrix, should be incorporated. However, to get a clear understanding of drug penetration, angiogenesis, tumor oxygenation, metastasis, and the impact of all physical aspects of a TME (e.g., increased ECM stiffness, confinement, and fluid flow), a 3D model should be developed within a microfluidic device or a lab-on-a-chip.

Although microfluidic devices have been extensively being used to simulate fluid flow that is present in tumors, they usually lack tumor heterogeneity and mechanical aspects of TME, such as tissue stiffness or compressive stress. Novel microfluidic devices that include different cell types allowing live cell imaging and *organoids-on-a-chip* platforms provide even more physiologically relevant setups for modeling the TME.^126,127^ Since TME has a dominant role in regulating tumor formation, progression, and metastasis, these strategies allow the study of cancer cell behavior under dynamic flow conditions, confinement, and increased ECM stiffness.^128,129^ Chen *et al.*, developed such a model composed of a micro-vessel wall, ECM matrix, and uniformly sized multicellular breast tumor spheroids (MCTS) for the evaluation of nanoparticle drug delivery systems, while a microplate reader was used to monitor cell viability *in situ*.^130^ In a more recent work, a microfluidic platform employing tumor spheroids embedded within a hydrogel was used to demonstrate the mechanical interdependency between the material properties of cancer cells and their surrounding matrix. By applying hydrostatic pressure to deform the hydrogel, and along with confocal imaging, this work revealed two distinct modes of cell growth depending on the elastic modulus of the surrounding spheroid hydrogel.^131^ Interstitial flow induced-shear stress, simulated within a similar microfluidic device, was able to mediate liver cancer cell-induced activation of normal fibroblasts to CAFs, through tumor-derived exosomes that activate the IGF2-PI3K signaling axis.^55^

However, for the microfluidic devices to fully restructure the 3D TME architecture and composition as well as to employ components of the vascular and immune system, the complexity of these experimental setups has been remarkably increased.^132–134^ For instance, a microfluidic device containing breast cancer cell spheroids co-cultured with macrophages, monocytes, and endothelial cells within 3D ECM in the presence of interstitial fluid was used to study tumor cell migration and invasion. It was shown that MDA-MB-231 spheroids promoted the transition of monocytes into tumor-associated macrophages (TAMs) through the colony-stimulating factor 1 (CSF-1). These activated macrophages further promoted angiogenesis via VEGF and tumor cell invasion.^135^ In a similar manner, a multi-organ-on-a-chip platform was employed to explore the interaction of NK cells with neuroblastoma cancer cells. In this platform, fluid flow-driven NK cells were examined for their ability to infiltrate a 3D alginate matrix to target the encapsulated tumor cell spheroids.^136^ Another novel 3D microfluidic device, employed to study the extravasation of breast cancer cells to bones, was consisted of cancer cells, human osteo-differentiated bone marrow-derived mesenchymal stem cells, and endothelial cells enabling the crosstalk between three different cell types in a bone-like environment.^137^ Recently, Kim *et al.* developed a unique microfluidic device to generate fluid flow from a channel consisting of cancer cells to a recipient channel with fibroblasts mimicking the tumor stroma. The fluid flow from the donor to the recipient channel carried secreted factors from cancer cells, which stimulated the activation of fibroblasts on the receiving side, as well as their migration. The expression of MMPs as well as CAF markers such as fibroblast activation protein alpha (FAPα), vimentin, and alpha-smooth muscle actin (α-SMA) were highly expressed in these migrating fibroblasts.^138^ Nevertheless, while these systems offer distinct advantages over the traditional platforms, their development is often time-consuming, costly, and challenging.

Recent efforts have been made toward automated approaches, such as 3D bioprinting. 3D bioprinting uses a computer-aided design that allows the deposition of living cells along with signaling molecules to generate highly controlled tissue engineered constructs.^139^ 3D bioprinted tumor constructs have characteristics like the *in vivo* tumor tissues, such as high growth rates of cancer cells, elevated invasiveness, angiogenesis, metastasis, and high resistance to anticancer drugs and have been developed for breast,^140,141^ pancreatic,^142,143^ and lung cancer.^144^ Although these models usually include ECM-mimicking matrices that are not capable of creating the diverse and dense stroma of highly desmoplastic tumors, such as pancreatic cancer, several types of self-assembling peptides have been developed for the *ex vivo* modeling of tissues with enhanced versatility.^145–147^ Improvements in collagen networks have also been made to better recapitulate the *in vivo* tissue microenvironments. A novel heterogeneous mesoscopic architecture, which can capture the *in vivo* tissue feature, was recently developed to study mesenchymal stem cell behavior.^148^ As current models are based on altering individual local properties of a collagen network, such as pore size and stiffness, Liu *et al.* developed a novel method of fabricating a thick collagen network by altering fibrillogenesis and gelation *in vitro*. Their results showed that generating a collagen network with spatial heterogeneity but maintaining a global homogeneity, resulted in different morphological features and migratory capabilities of breast cancer cells.^149^

### The promise of combining multi-omics with mechanically active organoids-on-a-chip

Apart from selecting the more suitable experimental setup, it is reasonable to consider that to study the effect of physical cues on cellular responses, it would be valuable to conduct a large-scale analysis (such as transcriptomics, proteomics, etc.) on a cell-type-specific basis, since traditional analytical assays, such as quantitative Real Time PCR (qPCR) or western blotting, usually provide limited results.

Toward these directions, breast cancer cells cultured as 3D spheroids growing in alginate-gelatin microcapsules were analyzed by RNA sequencing. Analysis of the results revealed that mechanical stress caused by the growth of the spheroids within confined conditions induced changes in the expression of genes implicated in cytoskeletal organization, cell cycle, DNA repair, migration, stemness, and resistance to anticancer drugs.^41^ In a similar manner, proteomic analysis of shear stress-induced ovarian cancer cells revealed alterations in the phosphorylation of proteins implicated in cell cycle, cell structure, and lipid metabolism.^150^ Analysis of RNA sequencing data and comparisons between control and compressed cells also revealed that autophagy-related genes are upregulated in compressed breast and pancreatic cancer cells through PI3K signaling axis activation.^151^ In 2022, we performed a large-scale proteomic assay (reverse phase protein array, RPPA) to show that mechanical compression applied on pancreatic cancer cells growing in a 2D setup activates a signaling cascade involving the p38 MAPK, and JNK to promote cytoskeleton remodeling, and cell migration through Rac1/c′dc42/Myosin II axis.^152^ We also provided evidence that mechanical stress inhibits cell cycle progression and apoptosis and promotes autophagy activation as stress mitigation pathways of cells under compression. In a more recent study, transcriptomics analysis of colorectal cancer cells grown as spheroids in stiff hydrogels revealed that the TRPV4 mechano-sensor is activated in response to mechanical stress to trigger PI3K/Akt signaling axis and upregulation of the heat shock protein 70 (HSP70).^153^ It is noteworthy that these studies were primarily centered on cancer cells subjected to a particular mechanical stimulus, and although they have contributed novel insights into the role of mechanical forces in tumor progression, their focus on singular mechanical cues limits the comprehensiveness of the findings. The integration of diverse cell types within the same *in vitro* tumor model, where all relevant mechanical cues—encompassing factors like ECM stiffness, interstitial fluid flow, and compressive forces—as well as the tumor vasculature should be considered. To achieve this, tumor organoids should be selected as an ideal tumor model to be incorporated into a microfluidic device followed by a large-scale analysis, such as proteomic assay at a single-cell resolution. When these data will be validated in patient tumor samples, this approach will enable the identification and characterization of mechanically induced biomarkers across different cell types (Fig. 3).

In fact, the first report using quantitative mass spectrometry proteomics to study organoids was published in 2015 by Boj *et al.*^124^ They established organoid models from murine and human pancreatic tissues, either normal or neoplastic, but instead of culturing them *in vitro,* they orthotopically implanted them in animal models and allowed them to progress to invasive pancreatic tumors. Then, transcriptomic and proteomic analysis followed by correlation with human patient samples revealed that nucleoporins are broadly upregulated in the neoplastic murine organoids.^124^ In 2017, Cristobal *et al.* performed a mass-spectrometry proteomic and transcriptomic analyses of human colon organoids derived from healthy and tumor tissues from different patients. They identified a common proteomic and genomic profile in the tumor samples, with each tumor organoid exhibiting a unique proteomic signature corresponding to each cancer patient.^154^ Similar analysis has been performed between different clonal cells of the same colorectal cancer patient using an organoid system to study how tumor heterogeneity impacts immunotherapy resistance. Their findings demonstrate diverse peptide presentation even within a single individual, suggesting potential for a multi-peptide vaccination targeting to increase the response of patients who exhibit small number of genetic mutations.^155^ A transcriptome-wide analysis conducted on breast cancer cell lines and CAFs revealed compression-induced alterations in the microRNA and mRNA profiles, which were associated with tumor progression.^156^ This study involved growing cells in a 3D environment in which compression was applied using alginate and iron beads embedded within an agarose matrix.^156^ In a more recent work, transcriptomic and bioinformatic analysis revealed that mechanical compression promotes tumor progression and chemoresistance of melanoma cancer cells.^157^ As microbiome research has gained much attention in the field of tumor biology,^158,159^ tumor organoids have been also used as a model system to study inflammatory responses and bacterial interaction. In a recent study Pleguezuelos-Manzano *et al.* exposed human intestinal organoids to genotoxic *Escherichia coli*, which produce a set of enzymes able to induce DNA damage in the cells. Followed by whole-genome sequencing before and after this exposure revealed a unique profile of mutations that were absent from organoids injected with the non-genotoxic bacteria, enhancing the promise of microbiome in cancer treatment.^160^

Taken together, tumor organoids-based applications and large-scale analyses are becoming more relevant and may advance personalized treatment;^126^ however, the success rate of tumor organoids is still limited, while the impact of mechanical aspects of TME has yet to be considered. Recent studies used an automated approach to produce patient-derived breast and colorectal cancer organoids, which also include T cells. This technology could be used as a high-throughput tool for guiding clinical decisions as they allow the clinical testing of several therapies including immunotherapies and provide a platform for drug development.^161,162^ Mechanically active organoids-on-a-chip platforms that consider mechanical forces, such as compression due to confinement and shear forces, have also been developed.^127,163^ However, while these devices have demonstrated their capabilities as state-of-the-art platforms, they have not yet been extensively employed to study cancer progression or to investigate the impact of mechanical factors on tumor growth and therapy responses. Instead, their primary focus has been on establishing the feasibility of creating such devices and proposing their potential as ideal models for studying mechanical cues.^164^ The concept of utilizing organoids-on-a-chip technology to uncover novel mechanically induced biomarkers during the distinct stages of tumor progression also holds a great promise for early detection of challenging-to-diagnose cancers like pancreatic cancer. This approach could also predict the response of mechanically driven tumors to currently available mechanotherapeutic strategies (extensively reviewed in Ref. 7) directly boosting clinical decisions.

## CONCLUSIVE REMARKS AND REMAINING CHALLENGES

The impact of physical forces on cellular responses is now well established, yet the precise mechanisms governing these responses *in vivo* remain elusive. This gap could play a major role in drug failures observed in clinical trials. Despite the valuable insights derived from conventional 2D and 3D experimental setups, their inability to replicate the architecture and composition of tumors is crucial. Thus, innovative methodologies, including microfluidic devices and organ-on-a-chip platforms, coupled with multi-omics approaches, hold the potential to comprehensively unravel the impact of physical forces on tumor progression and therapy resistance.

However, key challenges remain for the successful implementation of such devices *in vitro.*^165,166^ These challenges stem from the high variability in the fabrication methods of each device that reduces the reproduction and standardization of culturing cells on microchips. Also, to better recapitulate the *in vivo* microenvironment, multiple cell lines should be introduced on a microchip platform either seeded as a monolayer or growing as spheroids. However, cell handling and the integration of multiple cell types or co-culture systems, as well as the proper loading of 3D features on microchips, are difficult to achieve; thus, devices should be specialized with proper 3D culture chambers that, at the same time, could allow live cell monitoring in the microscale system. Also, maintaining the stability and viability of different cell populations within the same culture system, especially of those growing in organoids, over extended periods on microchips can be challenging, since factors such as evaporation, cross-contamination between the different cell types, and bacterial contamination need to be carefully considered.^167^

Regarding the simulation of mechanical stimuli on microchips, modeling shear stress conditions requires the use of microfluidic channels, which can be sensitive to clogging and fouling, while optimizing flow conditions or the level of solid stress to minimize their effect on cell survival and functionality is essential.^166^ Regarding cell isolation, it should be noted that microfluidic devices and microchips usually require small sample volumes, making it challenging to extract enough cells for downstream analysis, such as proteomics or transcriptomics. Scaling up microfluidic processes for high-throughput applications or ensuring that the microfluidic device can handle larger sample volumes without compromising the experimental success should be considered. Another critical aspect is how to isolate a specific cell type from an organoid or a spheroid containing multiple cell lines for a subsequent analysis. The development of strategies for selective cell capture, where specific cells of interest are isolated, while others are excluded, is critical as the number of the selected cells could be very low, and the possibility of cross-contamination is high. Finally, choosing the right combination of enzymes and buffers for organoid and spheroid dissociation, which can efficiently break down the extracellular matrix without harming the cells is a critical step to be considered to get enough quality and quantity of sample for subsequent analysis.

In conclusion, while the progress of *in vitro* experimental setups holds significant promise for exploring the mechanically driven tumor progression, it is crucial to focus on addressing and overcoming the challenges of these setups to establish physiologically relevant conditions, ultimately boosting the success rate of therapeutic strategies in clinical applications.

## Data Availability

Data sharing is not applicable to this article as no new data were created or analyzed in this study.
